# Chief Complaints, Underlying Diagnoses, and Mortality in Adult, Non-trauma Emergency Department Visits: A Population-based, Multicenter Cohort Study

**DOI:** 10.5811/westjem.2022.9.56332

**Published:** 2022-10-31

**Authors:** Michael Dan Arvig, Christian Backer Mogensen, Helene Skjøt-Arkil, Isik Somuncu Johansen, Flemming Schønning Rosenvinge, Annmarie Touborg Lassen

**Affiliations:** * Slagelse Hospital, Department of Emergency Medicine, Slagelse, Denmark; † University of Copenhagen, Department of Clinical Medicine, Copenhagen, Denmark; ‡ University of Southern Denmark, Department of Clinical Research, Odense, Denmark; § University Hospital of Southern Denmark, Department of Emergency Medicine, Aabenraa, Denmark; ¶ Clinical Institute, University of Southern Denmark, Research Unit for Infectious Diseases, Odense, Denmark; || University of Southern Denmark, Department of Regional Health Research, Odense, Denmark; # Odense University Hospital, Department of Infectious Diseases, Odense, Denmark; ** University of Southern Denmark, Odense University, Hospital, Open Patient data Explorative Network (OPEN), Odense, Denmark; †† Odense University Hospital, Department of Clinical Microbiology, Odense, Denmark; ‡‡ University of Southern Denmark, Research Unit of Clinical Microbiology, Odense, Denmark; §§ Odense University Hospital, Department of Emergency Medicine, Odense, Denmark

## Abstract

**Introduction:**

Knowledge about the relationship between symptoms, diagnoses, and mortality in emergency department (ED) patients is essential for the emergency physician to optimize treatment, monitoring, and flow. In this study, we investigated the association between symptoms and discharge diagnoses; symptoms and mortality; and we then analyzed whether the association between symptoms and mortality was influenced by other risk factors.

**Methods:**

This was a population-based, multicenter cohort study of all non-trauma ED patients ≥18 years who presented at a hospital in the Region of Southern Denmark between January 1, 2016–March 20, 2018. We used multivariable logistic regression to examine the association between symptoms and mortality adjusted for other risk factors.

**Results:**

We included 223,612 ED visits with a median patient age of 63 and even distribution of females and males. The frequency of the chief complaints at presentation were as follows: non-specific symptoms (19%); abdominal pain (16%); dyspnea (12%); fever (8%); chest pain (8%); and neurologic complaints (7%). Discharge diagnoses were symptom-based (24%), observational (hospital visit for observation or examination, 17%), circulatory (12%), or respiratory (12%). The overall 30-day mortality was 3.5%, with 1.7% dead within 0–7 days and 1.8% within 8–30 days. The presenting symptom was associated with mortality at 0–7 days but not with mortality at 8–30 days. Patients whose charts were missing documentation of symptoms (adjusted odds ratio [aOR] 3.5) and dyspneic patients (aOR 2.4) had the highest mortality at 0–7 days across patients with different primary symptoms. Patients ≥80 years and patients with a higher degree of comorbidity had increased mortality from 0–7 days to 8–30 days (aOR from 24.0 to 42.7 and 1.9 to 2.8, respectively).

**Conclusion:**

Short-term mortality was more strongly associated with patient-related factors than with the primary presenting symptom at arrival to the hospital.

## INTRODUCTION

A patient presents at a hospital with one or more chief complaints.^1^ The initial triage and work-up are primarily driven by the patient’s symptoms, whereas the patient’s final diagnosis and prognosis determine the subsequent evaluation and monitoring.^2^ Acute diagnostic decisions are based on symptoms, objective findings, and patient-related factors such as age, comorbidity, and lifestyle factors. Therefore, it is essential to have a systematic knowledge of the associations between complaints, diagnoses, and prognostic outcomes (eg, mortality, readmissions, and length of stay). Furthermore, this understanding can support the clinician in prioritizing resources and logistics in the emergency department (ED) and potentially prevent unsuspected deterioration.

Several studies have investigated the relationship between presenting symptoms and prognostic outcomes, but the studies are either too small,^3^–^5^ focus on specific patient categories,^6^ or specific symptoms.^7^ Other studies focus on the prehospital setting, where the approach to patients can differ from the hospital setting and not all patients are admitted.^8^,^9^ Thus, the current research is inadequate to establish generalizability for the attending emergency physician to handle the daily flow, crowding, and assessment of ED patients.

We conducted a population-based, multicenter cohort study among adult, non-trauma ED patients arriving at a hospital in the Region of Southern Denmark (RSD) from January 2016–March 2018. The research objectives were as follows: 1) describe the proportions of the most common symptoms and underlying diagnoses; 2) analyze the association between symptoms and mortality at 0–7 and 8–30 days; and 3) analyze whether other risk factors influenced the association between symptoms and mortality.

## METHODS

### Study Design and Setting

The study was a population-based, multicenter, dynamic cohort study of all non-trauma ED visits at hospitals in the RSD, covering a population of 1.2 million citizens.^10^ Data was collected from seven departments between January 1, 2016–March 20, 2018. The EDs provided 24-hour care and received patients referred from an ambulance or a primary care physician. In Denmark, referral is mandatory, and healthcare is tax-funded with free and equal access. We followed the Strengthening the Reporting of Observational Studies in Epidemiology Statement (STROBE) (Appendix 1).^11^

### Selection of Participants

We included non-trauma ED patients ≥18 years who arrived at a hospital. Registered visits without a unique Danish civil registration number (CRN) were excluded because the visits could not be linked to other national registers.

### Variables and Data Sources

Patients presenting at a hospital in the RSD were triaged by a nurse. Triage was done using the Danish Emergency Process Triage (DEPT).^12^,^13^ Based on presenting complaints and vital signs, DEPT categorizes the patient into five degrees: red (life-threatening); orange (critical); yellow (stable but potentially unstable); green (stable); and blue (unaffected). In addition, the same nurse registered the patient’s primary complaint from a limited number of predefined possibilities at arrival. In some instances, a patient could not be attached to a specific symptom and was instead categorized as having non-specific symptoms (eg, patients who were unable to express their complaints sufficiently). It is important to use non-specific symptoms as an exclusions category and not an operational definition; otherwise, it would require an exhaustive list of possible subcategories.^14^

**Population Health Research Capsule**

What do we already know about this issue?

*The relationship between symptoms, diagnoses, and prognosis of the ED patient is currently insufficient for handling flow, crowding, and acute patient care*.

What was the research question?

*What is the association between symptoms, diagnoses, and mortality in a cohort of adult, non-trauma patients arriving at an ED?*

What was the major finding of the study?

*Visits to the ED are often due to non-specific symptoms, and age and comorbidity are most strongly associated with mortality*.

How does this improve population health?

*Recognizing age and comorbidity as important risk factors in patient evaluation is essential to improve patient care and logistics in the ED*.

Information about the patient’s CRN, presenting symptoms, and triage level was drawn from the patient administrative system. Each patient visit was linked to the Danish Civil Registration System and the Danish National Patient Registry,^15^,^16^ from which we extracted data about gender, age, time of death, admission, and the discharge date, and the discharge diagnoses (based on *the International Classification of Diseases, 10th Rev*.) assigned by a physician. The Charlson Comorbidity Index (CCI) was calculated from the prior 10 years of diagnoses before the index date.^17^ According to clinical judgment and based on similar studies, we further divided CCI into three levels (0, 1, ≥2 points) according to the degree of comorbidity, and we grouped age into three subgroups (18–49, 50–79, and ≥80 years).^8^,^18^ Mortality was divided into 0–7 days and 8–30 days since the association between mortality and the degree of acuteness plus abnormal vital signs at admission is known to decrease after seven days.^17^ In patients with more than one admission registered, the date of the first admission was used as the index date for calculating the absolute and relative mortality.

### Statistical Methods

We summarized continuous variables as medians and interquartile range (IQR). Symptoms, diagnoses, and mortality were described as frequencies and percentages of the total sample. We tested the association between symptoms and mortality with a logistic regression model to obtain crude odds ratios (cOR) and adjusted odds ratios (aOR) for possible other risk factors (age, gender, CCI, time of arrival, and day of arrival). Patients were followed to death, emigration, or 30 days following index date, whichever came first. We handled missing values regarding symptoms as independent variables in a separate group. We performed a post-hoc analysis of mortality at 31–365 days to explore whether the general pattern was still present. All statistical analyses were done with STATA version 17.0 (StataCorp, College Station, TX).

### Ethics

The Danish Patient Safety Authority approved the study (identifier 3-3013-2272/1). The RSD permitted data storage (identifier 17/24904, amendment identifier 20/24502). All data were stored, secured, and managed according to the laws and regulations in the General Data Protection Regulation and the Danish Data Protection Act.^19^,^20^ According to the Act on Research Ethics Review of Health Research Projects, register-based studies do not require approval from the research ethics committee system.^21^

## RESULTS

### Characteristics of Study Subjects

**Figure 1:**
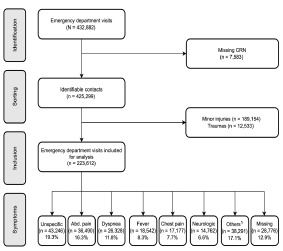
Flow diagram of the selection of adult non-trauma emergency department patients arriving at a hospital in the Region of Southern Denmark between 2016–2018, and the distribution of patients according to the most frequent chief symptoms at arrival. ^1^Other symptoms have an individual percentage < 3% and consists of the following: palpitations (5,107 [2.2%]), fainting (4,802 [2.1%]), gastrointestinal bleeding (4,407 [2.0%]), surgical abscess (3,016 [1.32%]), unconsciousness (2,964 [1.3%]), genital tract bleeding (2,915 [1.3%]), poisoning (2,554 [1.1%]), convulsions (2,525 [1.1%]), diarrhea or/and vomiting (2,027 [0.9%]), back pain (1,650 [0.7%]), headache (1,643 [0.7%]), allergy/anaphylaxis (964 [0.4%]), fall (887 [0.4%]), pain in the scrotum (884 [0.4%]), withdrawal (719 [0.3%]), dysphagia (377 [0.2%]), delirium (374 [0.2%]), cardiac arrest (189 [0.1%]), dizziness (165 [0.1%]), acute psychosis (130 [0.1%]), symptoms from the urinary tract (110 [0.1%]), peripheral edema (76 [0.0%]), high blood pressure (69 [0.0%]), and septic (19 [0.0%]). *Abd*, abdominal; *CRN*, civil registration number.

Of the 432,882 ED visits sampled, 7,583 without a CRN (foreign or immigrated patients) were excluded (Figure 1). We also removed trauma patients (12,533) and patients with minor injuries (189,154) from the analysis because they were handled by a trauma team or evaluated in a fast-track system. Thus, 223,612 ED visits were included in the final analysis. The median age was 63 years, with an even distribution of females and males (Table 1). Most patients arrived Monday–Friday during the day and were triaged yellow. About one third of the patients had one or more comorbidities.

### Main Results

The most frequent symptoms on arrival were non-specific (19.3%), abdominal pain (16.3%), dyspnea (11.8%), fever (8.3%), chest pain (7.7%), and neurological complaints (6.6%). Within these six symptom categories, patients did not vary regarding gender, age, and comorbidities. In 17.1% of the patients, other symptoms were present in <3% individually. The remaining 12.9% of the patients lacked documentation of their primary complaints. These patients differed from the other patients by missing triage, younger age, more females, and fewer comorbidities.

The most frequent discharge diagnoses were symptom-based diagnosis (24.0%), observational diagnosis (hospital visit for observation or examination [16.9%]), diseases of the circulatory system (11.9%), and respiratory system (11.7%) (Table 2). We also saw this general pattern between the different symptoms; however, differences were present regarding the organ-based diagnoses. The overall 30-day mortality was 3.5%, with mortality at 0–7-days and 8–30 days of 1.7% and 1.8%, respectively. The mortality at 31–365 days was 6.4%. Patients with dyspnea had the highest absolute mortality—overall and subdivided into mortality at 0–7 days and 8–30 days (Table 1).

**Figure 2:**
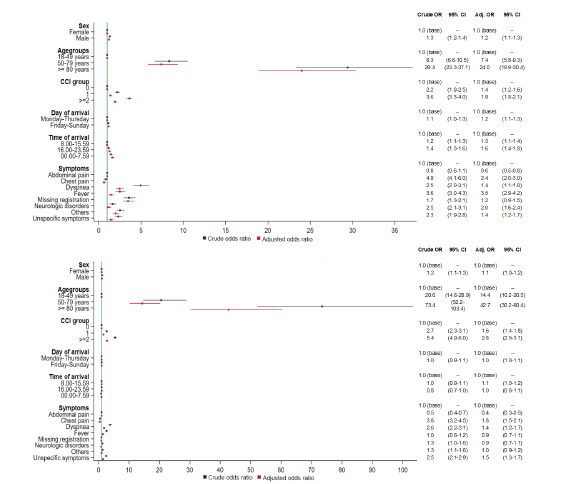
Odds ratios and adjusted odds ratios for the association between risk factors and mortality at 0–7 (A) and 8–30 days (B) among adult, non-trauma, emergency department patients arriving at a hospital in the Region of Southern Denmark between 2016–2018. *CCI*, Charlson Comorbidity Index.

Male gender, age ≥50 years, CCI ≥2, arrival on the weekend, arrival in the evening, and at night increased mortality at 0–7 days. However, only age (adjusted odds ratio [aOR] for 50–79 years and ≥80 years doubled) and CCI ≥2 (aOR increased 1.5-fold) remained significant strong risk factors for mortality at 8–30 days (Figure 2). The same pattern was seen in mortality at 31–365 days (Appendix 2) but with a decrease in the importance of age and CCI. Dyspneic patients (cOR 4.9) and patients lacking documentation of symptoms (cOR 3.6) had the highest mortality at 0–7 days. Adjusted for other risk factors, patients whose charts were missing documentation of symptoms (aOR 3.5) had the highest mortality at 0–7 days among the different presenting complaints.

## DISCUSSION

In this large cohort study, we found that patients most commonly arrived at the hospital with non-specific symptoms, abdominal pain, and dyspnea and were discharged with an observational- or a symptom-based diagnosis. Age and comorbidity were strong risk factors for mortality at 0–7 and 8–30 days, whereas the primary symptoms only had an association with short-term mortality.

Non-specific complaints were the most common reason for ED visits, with abdominal pain, dyspnea, fever, chest pain, and neurological complaints being the more specific reasons; an equal distribution has been shown in other countries and in the prehospital setting.^4^,^22^,^23^ Patients with non-specific complaints risk suffering severe conditions.^14^ These patients are typically time-consuming, and the workflow can be inefficient.^24^ The ED should consequently have protocols for handling this patient category to optimize daily practice and prevent adverse health outcomes.

Symptom- or observational-discharge diagnosis, as the most common, has also been seen throughout past decades in the prehospital setting, inpatient admissions from the ED, and emergency care visits.^25^,^26^ Several reasons could explain this: 1) a specific diagnosis could not be found; 2) other diagnostic procedures were necessary to establish a final diagnosis but were handled in an outpatient clinic afterward; or 3) the symptoms disappeared during admittance before a final diagnosis was established. Not receiving a final diagnosis has both patient- and clinician-oriented implications. The patients could become insecure about their health condition. From the clinician’s point of view, this might lead to overtesting and overtreatment.^27^,^28^ The physician, therefore, has a central role through clear communication with the patient to avoid this vicious circle.

Overall, older age and degree of comorbidity were associated with the highest mortality, also after adjusting for other risk factors. Most triage systems and early warning scores do not include age and comorbidities.^29^,^30^ Failure to recognize these risk factors could lead to undertriage and underscoring and, thus, potentially severe adverse events. In line with our results, the National Early Warning Score (NEWS) has been found in a recent international study to improve the prediction of in-hospital mortality when combined with age.^31^ On the contrary, the type of symptom and the time and day of arrival only have an impact in the short run. Therefore, the presenting symptoms might, combined with age and comorbidity, provide valuable prognostic information during the patient’s initial evaluation and in prioritizing among patients.

Patients with missing registration of symptoms had the highest aOR for mortality at 0–7-days across the different primary symptoms. An explanation for the missing record of symptoms could be work pressure, the circumstances surrounding the patient, the logistics in general in the ward at the given time, or an unstable patient unable to express their chief complaint. The high aOR for mortality of these patients could suggest some degree of urgency and underlying deterioration. One study of ED patients who were missing values for vital signs found an association with short-term mortality, indicating that values were not missing at random.^18^ Patients with dyspnea had the highest OR for mortality at 0–7 days among patients with specific symptoms. However, the association was less pronounced in the adjusted analysis, implying that other risk factors contributed to the mortality.^32^,^33^

## LIMITATIONS

The first limitation to consider is that patients with missing CRN were excluded, which could have introduced selection bias. However, these patients constituted only <2% of the total sample. Second, when missing values appeared regarding symptoms we handled them as an independent variable in the multivariable analysis to show that missing data were not missing at random but associated with high short-term mortality, thereby providing valuable information. Third, the type of symptom documented at the patient’s arrival depended on the healthcare worker on shift at the given time. However, the categorization of symptoms was based on a limited number of options to provide consistency in the registration. Fourth, comorbidity was based on CCI extracted at the hospital level and potentially could have been missing diagnoses treated by the general practitioner.

## CONCLUSION

Recognizing age and comorbidity is essential in the primary evaluation of ED patients and subsequent monitoring. Future research in triage systems and early warning scores should incorporate these factors to improve clinical outcomes and guide clinicians in their daily work.

## Supplementary Information





## Figures and Tables

**Table 1:** Characteristics of the adult non-trauma emergency department patients arriving at a hospital in the Region of Southern Denmark between 2016–2018.

	Total	Non-specific symptoms	Abdominal pain	Dyspnea	Fever	Chest pain	Neurologic complaints	Others^1^	Missing registration
Total, N (%)	223,612 (100.0)	43,246 (19.3)	36,490 (16.3)	26,328 (11.8)	18,542 (8.3)	17,177 (7.7)	14,762 (6.6)	38,291 (17.1)	28,776 (12.9)
Gender									
Female	115,902 (51.8)	21,745 (50.3)	21,091 (57.8)	13,732 (52.2)	8,694 (46.9)	7,976 (46.4)	7,480 (50.7)	19,394 (50.6)	15,790 (54.9)
Male	107,710 (48.2)	21,501 (49.7)	15,399 (42.2)	12,596 (47.8)	9,848 (53.1)	9,201 (53.6)	7,282 (49.3)	18,897 (49.4)	12,986 (45.1)
Age, years, median (IQR)	63 (44–77)	68 (51–79)	52 (34–70)	72 (61–81)	70 (54–80)	61 (48–74)	66 (50–77)	60 (39–75)	54 (33–71)
Age groups (years)									
18–49	69,778 (31.2)	10,238 (23.7)	16,965 (46.5)	3,553 (13.5)	3,867 (20.9)	4,822 (28.1)	3,504 (23.7)	14,114 (36.9)	12,715 (44.2)
50–79	110,527 (49.4)	22,249 (51.4)	15,625 (42.8)	14,880 (56.5)	9,809 (52.9)	9,734 (56.7)	8,307 (56.3)	17,273 (45.1)	12,650 (44.0)
≥80	43,307 (19.4)	10,759 (24.9)	3,900 (10.7)	7,895 (30.0)	4,866 (26.2)	2,621 (15.3)	2,951 (20.0)	6,904 (18.0)	3,411 (11.9)
Charlson Comorbidity Index									
0	136,978 (61.3)	24,626 (56.9)	25,956 (71.1)	8,841 (33.6)	9,187 (49.5)	11,904 (69.3)	10,371 (70.3)	25,865 (67.5)	20,228 (70.3)
1	28,101 (12.6)	5,191 (12.0)	3,423 (9.4)	7,405 (28.1)	2,515 (13.6)	1,854 (10.8)	1,388 (9.4)	3,575 (9.3)	2,750 (9.6)
≥2	58,533 (26.2)	13,429 (31.1)	7,111 (19.5)	10,082 (38.3)	6,840 (36.9)	3,419 (19.9)	3,003 (20.3)	8,851 (23.1)	5,798 (20.1)
Triage level^2^									
Red (life-threatening)	5,966 (2.7)	785 (1.8)	222 (0.6)	1,559 (5.9)	426 (2.3)	165 (1.0)	586 (4.0)	1,547 (4.0)	676 (2.3)
Orange (unstable)	40,397 (18.1)	6,187 (14.3)	3,378 (9.3)	6,411 (24.4)	2,936 (15.8)	6,684 (38.9)	3,653 (24.7)	8,840 (23.1)	2,308 (8.0)
Yellow (potentially unstable))	76,806 (34.3)	14,390 (33.3)	18,177 (49.8)	11,094 (42.1)	9,389 (50.6)	3,440 (20.0)	4,285 (29.0)	12,445 (32.5)	3,586 (12.5)
Green (stable)	66,522 (29.7)	17,491 (40.4)	12,774 (35.0)	6,041 (22.9)	5,110 (27.6)	5,137 (29.9)	5,052 (34.2)	11,709 (30.6)	3,208 (11.1)
Missing	33,921 (15.2)	4,393 (10.2)	1,939 (5.3)	1,223 (4.6)	681 (3.7)	1,751 (10.2)	1,186 (8.0)	3,750 (9.8)	18,998 (66.0)
Day of arrival									
Monday–Thursday	137,412 (61.5)	26,750 (61.9)	22,275 (61.0)	15,981 (60.7)	11,099 (59.9)	10,429 (60.7)	9,181 (62.2)	22,409 (58.5)	19,288 (67.0)
Friday–Sunday	86,200 (38.5)	16,496 (38.1)	14,215 (39.0)	10,347 (39.3)	7,443 (40.1)	6,748 (39.3)	5,581 (37.8)	15,882 (41.5)	9,488 (33.0)
Time of arrival									
8 AM – 3.59 PM	122,862 (54.9)	24,627 (56.9)	18,959 (52.0)	13,910 (52.8)	9,687 (52.2)	8,143 (47.4)	8,690 (58.9)	20,875 (54.5)	17,971 (62.5)
4 PM – 11.59 PM	71,615 (32.0)	14,467 (33.5)	12,446 (34.1)	8,235 (31.3)	6,883 (37.1)	5,524 (32.2)	4,872 (33.0)	12,194 (31.8)	6,994 (24.3)
12 AM–7.59 AM	29,135 (13.0)	4,152 (9.6)	5,085 (13.9)	4,183 (15.9)	1,972 (10.6)	3,510 (20.4)	1,200 (8.1)	5,222 (13.6)	3,811 (13.2)
Mortality^3^									
0–7-days mortality	1,991 (1.7)	357 (1.6)	147 (0.7)	389 (3.4)	159 (1.7)	60 (0.6)	107 (1.2)	372 (1.8)	400 (2.5)
8–30-days mortality	2,125 (1.8)	571 (2.6)	220 (1.1)	448 (3.9)	246 (2.7)	55 (0.5)	122 (1.3)	296 (1.4)	167 (1.0)

Values are numbers (%), unless otherwise noted.

1 Other symptoms have an individual percentage < 3% and consists of the following symptoms: palpitations (5,107 [2.2%]), fainting (4,802 [2.1%]), gastrointestinal bleeding (4,407 [2.0%]), surgical abscess (3,016 [1.32%]), unconsciousness (2,964 [1.3%]), genital tract bleeding (2,915 [1.3%]), poisoning (2,554 [1.1%]), con-vulsions (2,525 [1.1%]), diarrhea or/and vomiting (2,027 [0.9%]), back pain (1,650 [0.7%]), headache (1,643 [0.7%]), allergy/anaphylaxis (964 [0.4%]), fall (887 [0.4%]), pain in the scrotum (884 [0.4%]), withdrawal (719 [0.3%]), dysphagia (377 [0.2%]), delirium (374 [0.2%]), cardiac arrest (189 [0.1%]), dizziness (165 [0.1%]), acute psychosis (130 [0.1%]), symptoms from the urinary tract (110 [0.1%]), peripheral edema (76 [0.0%]), high blood pressure (69 [0.0%]), and septic (19 [0.0%]).

2 Triage is categorized into levels depending on the patient’s presenting complaint(s) and the vital signs.

3 Mortality is calculated for the first visit at a hospital because some patients were admitted several times during the inclusion period.

*IQR*, interquartile range; *ED*, emergency department.

**Table 2:** Distribution of discharge diagnoses according to the major groups of the International Classification of Diseases, 10th Rev., allocated between the most common symptoms for adult, non-trauma, emergency department patients arriving at a hospital in the Region of Southern Denmark between 2016–2018.

	Total	Non-specific symptoms	Abdominal pain	Dyspnea	Fever	Chest pain	Neurologic complaints	Others^1^	Missing registration
Total, N (%)	223,612 (100.0)	43,246 (19.3)	36,490 (16.3)	26,328 (11.8)	18,542 (8.3)	17,177 (7.7)	14,762 (6.6)	38,291 (17.1)	28,776 (12.9)
A00–B99 Certain infectious and parasitic diseases	9,598 (4.3)	2,217 (5.1)	705 (1.9)	1,034 (3.9)	3,595 (19.4)	124 (0.7)	169 (1.1)	1,330 (3.5)	424 (1.5)
C00–D89 Neoplasm and diseases of the blood and the immune system	4,979 (2.2)	1,722 (4.0)	645 (1.8)	454 (1.7)	410 (2.2)	88 (0.5)	280 (1.9)	798 (2.1)	582 (2.0)
E00–90 Endocrine, nutritional and metabolic diseases	7,413 (3.3)	4,629 (10.7)	268 (0.7)	375 (1.4)	471 (2.5)	120 (0.7)	190 (1.3)	1,061 (2.8)	299 (1.0)
F00–99 Mental and behavioral disorders	5,455 (2.4)	1,523 (3.5)	88 (0.2)	181 (0.7)	149 (0.8)	136 (0.8)	203 (1.4)	2,666 (7.0)	509 (1.8)
G00–99 Diseases of the nervous system	6,504 (2.9)	790 (1.8)	32 (0.1)	83 (0.3)	137 (0.7)	52 (0.3)	3,383 (22.9)	1,477 (3.9)	550 (1.9)
I00–99 Diseases of the circulatory system	26,523 (11.9)	5,152 (11.9)	497 (1.4)	3,221 (12.2)	660 (3.6)	3,184 (18.5)	2,864 (19.4)	4,680 (12.2)	6,265 (21.8)
J00–99 Diseases of the respiratory system	26,271 (11.7)	3,055 (7.1)	496 (1.4)	13,774 (52.3)	5,382 (29.0)	686 (4.0)	237 (1.6)	1,444 (3.8)	1,197 (4.2)
K00–93 Diseases of the digestive system	22,392 (10.0)	2,590 (6.0)	12,825 (35.1)	316 (1.2)	757 (4.1)	504 (2.9)	59 (0.4)	4,275 (11.2)	1,066 (3.7)
L00–99 Diseases of the skin	3,040 (1.4)	586 (1.4)	178 (0.5)	38 (0.1)	256 (1.4)	13 (0.1)	8 (0.1)	1,630 (4.3)	331 (1.2)
M00–99 Diseases of the muscoloskeletal system and connective tissue	5,755 (2.6)	2,020 (4.7)	258 (0.7)	196 (0.7)	517 (2.8)	334 (1.9)	183 (1.2)	1,314 (3.4)	933 (3.2)
N00–99 Diseases of the genitourinary system	11,417 (5.1)	2,477 (5.7)	2,720 (7.5)	499 (1.9)	2,753 (14.8)	128 (0.7)	212 (1.4)	1,849 (4.8)	779 (2.7)
O00–99 Pregnancy, childbirth and puerperium	2,811 (1.3)	382 (0.9)	340 (0.9)	10 (0.0)	79 (0.4)	6 (0.0)	0 (0.0)	1,123 (2.9)	871 (3.0)
R00–99 Symptoms, signs and clinical and laboratory findings, not elsewhere classified	53,740 (24.0)	9,336 (21.6)	12,859 (35.2)	4,300 (16.3)	2,012 (10.9)	6,454 (37.6)	4,391 (29.7)	9,422 (24.6)	4,966 (17.3)
Z00–99 Admittance for observation or examination	37,714 (16.9)	6,767 (15.6)	4,579 (12.5)	1,847 (7.0)	1,364 (7.4)	5,348 (31.1)	2,583 (17.5)	5,222 (13.6)	10,004 (34.8)

1 Other symptoms have an individual percentage < 3% and consists of the following symptoms: palpitations (5,107 [2.2%]), fainting (4,802 [2.1%]), gastrointestinal bleeding (4,407 [2.0%]), surgical abscess (3,016 [1.32%]), unconsciousness (2,964 [1.3%]), genital tract bleeding (2,915 [1.3%]), poisoning (2,554 [1.1%]), convulsions (2,525 [1.1%]), diarrhea or/and vomiting (2,027 [0.9%]), back pain (1,650 [0.7%]), headache (1,643 [0.7%]), allergy/anaphylaxis (964 [0.4%]), fall (887 [0.4%]), pain in the scrotum (884 [0.4%]), withdrawal (719 [0.3%]), dysphagia (377 [0.2%]), delirium (374 [0.2%]), cardiac arrest (189 [0.1%]), dizziness (165 [0.1%]), acute psychosis (130 [0.1%]), symptoms from the urinary tract (110 [0.1%]), peripheral edema (76 [0.0%]), high blood pressure (69 [0.0%]), and septic (19 [0.0%]).

*ED*, emergency department
