# Supplementary material for: Chief Complaints, Underlying Diagnoses, and Mortality in Adult, Non-trauma Emergency Department Visits: A Population-based, Multicenter Cohort Study
Source: West J Emerg Med. 2022 Oct 31;23(6):855–63. doi: 10.5811/westjem.2022.9.56332 (PMC9683768; doi:10.5811/westjem.2022.9.56332)
Supplement: Supplementary file 2 [file wjem-23-855-s002.pdf]

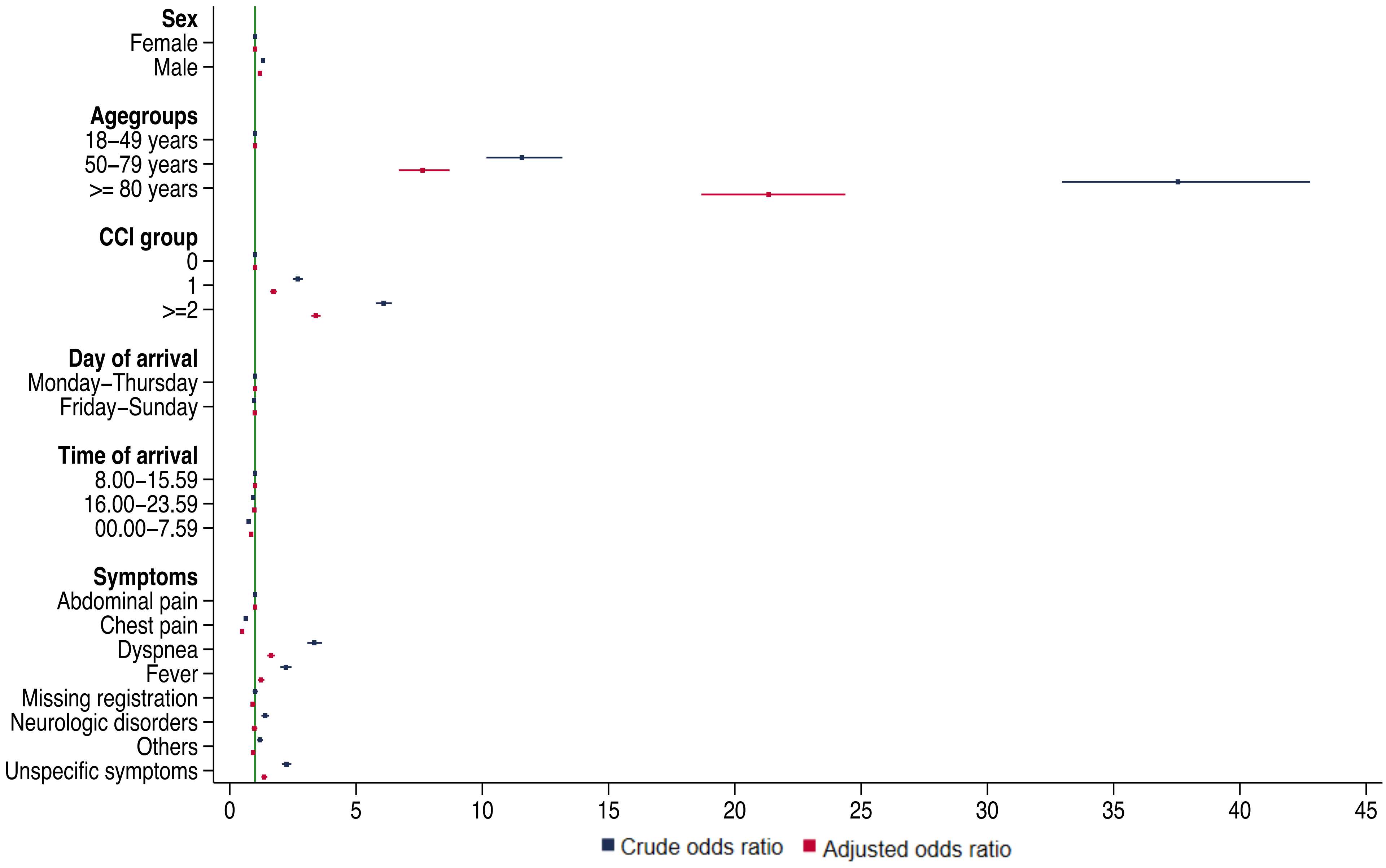

Crude OR	95% CI	Adj. OR	95% CI
1.0 (base)	–	1.0 (base)	–
1.3	(1.3-1.4)	1.2	(1.1-1.3)
1.0 (base)	–	1.0 (base)	–
11.6	(10.2-13.2)	7.6	(6.7-8.7)
37.5	(33.0-42.8)	21.3	(18.7-24.4)
1.0 (base)	–	1.0 (base)	–
2.7	(2.5-2.9)	1.8	(1.6-1.9)
6.1	(5.8-6.4)	3.4	(3.2-3.6)
1.0 (base)	–	1.0 (base)	–
1.0	(0.9-1.0)	1.0	(0.9-1.0)
1.0 (base)	–	1.0 (base)	–
0.9	(0.9-1.0)	1.0	(0.9-1.0)
0.7	(0.7-0.8)	0.8	(0.8-0.9)
1.0 (base)	–	1.0 (base)	–
0.6	(0.5-0.7)	0.5	(0.4-0.6)
3.3	(3.1-3.7)	1.6	(1.5-1.8)
2.2	(2.0-2.4)	1.2	(1.1-1.4)
1.0	(0.9-1.1)	0.9	(0.8-1.0)
1.4	(1.3-1.6)	1.0	(0.9-1.1)
1.2	(1.1-1.3)	0.9	(0.8-1.1)
2.2	(2.1-2.4)	1.4	(1.3-1.5)

Appendix 1. Crude and adjusted odds ratios for the association between risk factors and 31-356-day mortality among acute non-trauma adult patients arriving at a hospital in the Region of Southern Denmark between 2016-2018.
